# Supplementary material for: Extraction of clinical data on major pulmonary diseases from unstructured radiologic reports using a large language model
Source: PLoS One. 2024 Nov 25;19(11):e0314136. doi: 10.1371/journal.pone.0314136 (PMC11588275; doi:10.1371/journal.pone.0314136)
Supplement: S1 Table — (DOCX) [file pone.0314136.s001.docx]

**SUPPORTING INFORMATION**

**Extraction of clinical data on major pulmonary diseases from unstructured radiologic reports using a large language model**

Hyung Jun Park^1*^, Jin-Young Huh^2*^, Ganghee Chae^3†^, Myeong Geun Choi^4†^

^1^ Division of Pulmonary and Critical Care Medicine, Department of Internal Medicine, Gumdan Top Hospital, Incheon, Korea

^2^ Division of Pulmonary, Allergy and Critical Care Medicine, Department of Internal Medicine, Chung-Ang University Gwangmyeong Hospital, Gwangmyeong, Korea

^3^ Division of Pulmonary and Critical Care Medicine, Department of Internal Medicine, Ulsan University Hospital, University of Ulsan College of Medicine, Ulsan, Korea

^4^ Division of Pulmonary and Critical Care Medicine, Department of Internal Medicine, Mokdong Hospital, College of Medicine, Ewha Womans University, Seoul, Korea

^*^ These authors contributed equally to this study.

^†^**Corresponding Authors:**

**Myeong Geun Choi, MD, PhD**

Division of Pulmonary and Critical Care Medicine, Mokdong Hospital, College of Medicine, Ewha Womans University, 1071 Anyangcheon-ro, Yangcheon-gu, Seoul 07985, South Korea

Tel: +82-2-2650-5417

Fax: +82-2-2650-5272

E-mail: [cmkcmk1006@gmail.com](mailto:cmkcmk1006@gmail.com)

**Ganghee Chae, MD**

Division of Respiratory and Critical Care Medicine, Department of Internal Medicine, Ulsan University Hospital, University of Ulsan College of Medicine, 25 Daehakbyeongwon-ro, Dong-gu, Ulsan 44033, South Korea

Tel: +82-52-250-7029

Fax: +82-52-250-7048

E-mail: ganghee@uuh.ulsan.kr

**S1 Table. System and user prompts for extracting pulmonary outcomes from radiologic reports.**

The system prompted the model to detect the presence or absence of seven specific pulmonary diseases. In the user prompt, a verbatim radiologic report, such as a computed tomography (CT) scan or radiography (X-ray) report, is inserted instead of the "{report}" placeholder for analysis.

| System prompt | Extract the presence of diseases from the radiologic report provided below:  Please format the extracted information in JSON as follows:    "Pneumonia": "yes/no",  "Interstitial Lung Disease": "yes/no",  "Active Tuberculosis": "yes/no",  "Pulmonary Edema": "yes/no",  "Pleural Effusion": "yes/no",  "Lung Cancer": "yes/no",  "Emphysema": "yes/no" |
| --- | --- |
| User prompt | This is the radiologic report: {report} |
