# Supplementary material for: Extraction of clinical data on major pulmonary diseases from unstructured radiologic reports using a large language model
Source: PLoS One. 2024 Nov 25;19(11):e0314136. doi: 10.1371/journal.pone.0314136 (PMC11588275; doi:10.1371/journal.pone.0314136)
Supplement: S2 Table — (DOCX) [file pone.0314136.s002.docx]

**S2 Table. Fleiss’s Kappa value for interobserver agreement among pulmonologists.**

| Diseases | Chest radiography | | | | | Computed tomography | | | | |
| --- | --- | --- | --- | --- | --- | --- | --- | --- | --- | --- |
|  | Fleiss's Kappa | 95% CI | | z-Statistic | P | Fleiss's Kappa | 95% CI | | z-Statistic | P |
| Pneumonia | 0.735 | 0.700 | 0.771 | 38.2 | < 0.001 | 0.789 | 0.754 | 0.820 | 41.0 | < 0.001 |
| Interstitial lung disease | 0.755 | 0.668 | 0.821 | 39.2 | < 0.001 | 0.761 | 0.703 | 0.813 | 39.5 | < 0.001 |
| Active pulmonary tuberculosis | 0.384 | 0.264 | 0.535 | 20.0 | < 0.001 | 0.556 | 0.483 | 0.643 | 28.9 | < 0.001 |
| Pulmonary edema | 0.727 | 0.656 | 0.781 | 37.8 | < 0.001 | 0.870 | 0.795 | 0.922 | 45.2 | < 0.001 |
| Pleural effusion | 0.913 | 0.886 | 0.937 | 47.5 | < 0.001 | 0.864 | 0.832 | 0.889 | 44.9 | < 0.001 |
| Lung cancer | 0.740 | 0.677 | 0.796 | 38.5 | < 0.001 | 0.797 | 0.761 | 0.835 | 41.4 | < 0.001 |
| Emphysema | 0.941 | 0.908 | 0.966 | 48.9 | < 0.001 | 0.867 | 0.834 | 0.895 | 45.1 | < 0.001 |

95% CI: 95% confidence interval
