# Supplementary material for: Extraction of clinical data on major pulmonary diseases from unstructured radiologic reports using a large language model
Source: PLoS One. 2024 Nov 25;19(11):e0314136. doi: 10.1371/journal.pone.0314136 (PMC11588275; doi:10.1371/journal.pone.0314136)
Supplement: Attachment — Submitted filename: response_letter_241028.docx [file pone.0314136.s004.docx]

October 27, 2024

Dear Editor:

We greatly appreciate the valuable comments provided for our manuscript entitled “Extraction of clinical data on major pulmonary diseases from unstructured radiologic reports using a large language model.”

We are certain that the valuable suggestions from the reviewers have helped improve the quality of the manuscript. We have revised the manuscript according to the reviewers’ recommendations. The revised text has been indicated in blue font. Our point-by-point responses to all comments have been included below.

We hope that the revised manuscript is now suitable for publication in *PLOS One* as a Research Article.

Sincerely,

Myeong Geun Choi, MD, PhD

Division of Pulmonary and Critical Care Medicine, Mokdong hospital, College of Medicine, Ewha Womans University, 1071 Anyangcheon-Ro, Yangcheon-gu, Seoul, 07985, Korea

Tel: +82-2-2650-5417

Fax: +82-2-2650-5272

E-mail: cmkcmk1006@gmail.com

Ganghee Chae, MD

Division of Respiratory and Critical Care Medicine, Department of Internal Medicine, Ulsan University Hospital, University of Ulsan College of Medicine, 25 Daehakbyeongwon-ro, Dong-gu, Ulsan, 44033, South Korea

Tel: +82-52-250-7029

Fax: +82-52-250-7048

E-mail: [ganghee@uuh.ulsan.kr](mailto:ganghee@uuh.ulsan.kr)

**Editor:**

**Hard Recommendations (Must Address Before Acceptance):**

**Q1. Handling of Medical Abbreviations and Acronyms**:
The manuscript should explicitly discuss whether and how the LLMs differentiated between medical abbreviations (e.g., “PNA” for pneumonia) and their full forms. This is crucial for understanding the model’s capability in interpreting radiologic reports where abbreviations are commonly used.

**Reply:** Thank you for raising this important question about the language models’ ability to handle abbreviations and spelled-out words. We would like to clarify that the LLMs used in our study (Gemini Pro 1.0, GPT-3.5, and GPT-4) are indeed capable of understanding and differentiating between abbreviations and their full forms in context.

These models are trained on vast amounts of text data, including medical literature, which enables them to recognize common medical abbreviations like PNA for pneumonia and TB for tuberculosis, as well as their spelled-out versions. The models interpret these terms within the context of the entire radiologic report, allowing accurate inferences to be made regardless of whether abbreviations or full terms are used. We did not perform any specific preprocessing to standardize acronym usage, as the models demonstrated the ability to naturally handle this variation. This capability is part of the models’ strength in processing natural language as it appears in real-world medical reports, contributing to their effectiveness in our study.

**Changes in the text:** Lines 303–310

*“Furthermore, the LLM interpreted medical abbreviations within the context of the entire radiologic report, making inferences regardless of whether abbreviations or full terms were used. For instance, GPT-4 correctly recognized all instances of “TB” as “tuberculosis” in the reports. We did not perform any specific preprocessing to standardize acronym usage, as the models demonstrated the ability to handle this variation naturally. These capabilities are part of the models’ strength in processing natural language as it appears in real-world medical reports, contributing to its effectiveness in our study.”*

**Q2. Addressing Clinical Uncertainty**:
The manuscript must address how the LLMs handled clinical uncertainty present in radiologic reports, such as ambiguous diagnoses (e.g., “emphysema vs. COPD” or “lung cancer vs. metastasis”). A discussion on how the models interpreted these uncertainties and whether they required clinical correlation should be included.

**Reply:** We appreciate this valuable comment regarding how LLMs handle clinical uncertainty in radiologic reports. The models we used were pre-trained on various medical literature and seem to avoid making judgments on items with clinical uncertainty, that is, items in the radiologic report lacking definitive evidence for diagnosis. For example, upon reviewing the decisions made by GPT-4, it did not classify “developing and increasing in size of heterogeneously enhancing nodules in the right lower lobe“ as lung cancer on a CT scan of a patient with breast cancer. In a case where the report mentioned the possibility of chronic obstructive pulmonary disease, a condition where emphysema may appear in radiologic findings but is not essential for diagnosis, in a patient with chronic bronchitis, GPT-4 did not classify the finding as emphysema.

**Changes in the text:** Lines 295–303

*“On the other hand, in some cases, it appeared that the LLM correlated diagnostic criteria with the context of the radiologic report and avoided making judgments on items with clinical uncertainty, that is, items in the report lacking definitive evidence for diagnosis. For example, upon reviewing the decisions made by GPT-4, it did not classify “developing and increasing in size of heterogeneously enhancing nodules in the right lower lobe” as lung cancer on a CT scan of a patient with breast cancer. In a case where the report mentioned the possibility of chronic obstructive pulmonary disease, a condition where emphysema may appear in radiologic findings but is not essential for diagnosis [24], in a patient with chronic bronchitis, GPT-4 did not classify the finding as emphysema.”*

**Q3. Interobserver Pulmonologist Qualifications**:
The qualifications of the pulmonologists involved in setting the gold standard need to be clearly stated. This includes their experience, training, and roles within their institutions. Additionally, the manuscript should discuss whether the LLMs accounted for differences between trainee and attending radiologist reads, which could impact the results.

**Reply:** Thank you for your valuable comment. The three pulmonologists who performed the labeling have over 10 years each of experience as physicians, including more than 5 years each of clinical experience as pulmonologists. All three hold assistant professorships at university hospitals. GC’s clinical and research areas are tuberculosis (mycobacterial disease), lung cancer, and interventional pulmonology. MGC specializes in lung cancer and interventional pulmonology, and J-YH’s areas of expertise are lung cancer and interstitial lung disease. These details have been added to the Methods section.

**Changes in the text:** Lines 118–131

*“The following outcomes were defined: pneumonia, interstitial lung disease (ILD), active pulmonary tuberculosis (TB), pulmonary edema, pleural effusion, lung cancer, and emphysema. On CT scans, interstitial lung abnormalities were identified as ILD, and lung abscesses were classified as pneumonia. The complete resolution and improvement were recorded as absent, whereas interval improvement and decreases were recorded as present. On chest radiographs, pulmonary congestion was assessed for edema, and interstitial pneumonia was categorized as ILD. Three pulmonology specialists, GC, MGC, and J-YH, each with over a decade of experience as practicing physicians including more than 5 years each of clinical experience in pulmonology independently reviewed the reports and labeled the presence or absence of seven diseases. All three hold assistant professorships at university hospitals with clinical and research expertise. GC’s clinical and research areas include tuberculosis (mycobacterial disease), lung cancer, and interventional pulmonology; MGC specializes in lung cancer and interventional pulmonology; and J-YH’s areas of expertise are lung cancer and interstitial lung disease. The gold standard was established as an agreement among at least two labelers.”*

**Q4. Error Rate of GPT-4**:
The reported error rate of 0% for GPT-4 in generating JSON formats seems implausible. The authors should re-examine this claim, providing a more nuanced explanation of what “error-free” means in this context and whether any minor or non-critical errors were overlooked.

**Reply:** We appreciate the astute observation regarding the reported 0% error rate for GPT-4 in generating JSON formats. We acknowledge that this result warrants further explanation and have added clarification to our Methods and Discussion sections.

In our study, we defined “error-free“ JSON formats as outputs that strictly adhered to the specified structure, including all seven disease fields with appropriate “yes” or “no” values, without any syntactical errors or missing information. We have added this definition to our Discussion section for clarity. While GPT-4 achieved perfect structural accuracy in our dataset, we acknowledge that this does not reflect potential semantic errors in disease classification, which are separately addressed in our performance analysis.

**Changes in the text:** Lines 325–330

*“Although all AI models demonstrated strong agreement with human annotators in terms of performance, notable discrepancies were observed in their stability when generating JSON formats. GPT-4 showcased exceptional stability, flawlessly producing error-free JSON formats on its first attempt. Error-free JSON formats refer to outputs that strictly adhered to the specified structure, including all seven disease fields with appropriate “yes” or “no” values, without any syntactical errors or missing information.”*

**Q5. Introduction of JSON Format**:
The manuscript introduces the concept of JSON format without prior explanation, which may confuse readers. The authors should include a brief introduction to JSON formatting, particularly explaining its relevance and use in the study.

**Reply:** We thank the reviewer for pointing out the need for a clearer introduction to JSON formatting in our manuscript. We agree that this is an important aspect of our methodology that requires explanation for readers who may be unfamiliar with the concept.

To address this, we have added a brief explanation of JSON formatting and its relevance to our study in the Statistical Analysis section of the Methods. This addition provides context for our use of JSON and explains its role in evaluating the models’ performance.

**Changes in the text:** Lines 150–156

*“Additionally, the accuracy of each label was evaluated against the gold standard using these metrics, stratified by disease and hospital, to determine the accuracy and appropriateness of human annotation. Interobserver agreement among the labelers was assessed by calculating the Fleiss kappa value for each disease. The output of each model was evaluated for adherence to the specified JSON format, which included the correct structure and presence of all seven disease fields. Errors in JSON formatting were recorded and analyzed to assess the models’ ability to consistently produce structured output.”*

**Q6. Generalizability of Results**:
The study’s results are based on radiologic reports from Korean institutions, which may not generalize to other regions with different reporting styles. The manuscript should discuss this limitation and suggest future validation on more diverse datasets, including those from different languages and cultural contexts. The style of English usage in Korea may not be generalized elsewhere (especially in native English speaking countries). This remains a potential limitation of this study and should be discussed, especially when LLMs such as ChatGPT 4 seems to have heterogeneous performance in language translations in resource poor languages, as demonstrated in this recent study *https://www.mdpi.com/2075-4426/14/9/923*

**Reply:** Thank you for pointing out the potential limitation regarding external validation of our study. We acknowledge that our study utilized radiologic reports written by Korean radiologists, which may limit its generalizability to other regions where reporting styles and medical practices may differ. As suggested, future studies could benefit from validating the findings using datasets from different countries and linguistic backgrounds to determine the models’ performance across various contexts. We have added this point to the discussion of our study’s limitations.

**Changes in the text:** Lines 346–356

*“Moreover, this research utilized radiologic reports written by Korean radiologists, which may limit its generalizability to regions with different reporting styles and medical practices [28]. Future studies could benefit from validating these findings using datasets from other countries and linguistic backgrounds to assess the models’ performance across diverse contexts. Lastly, the models’ performance in this study may have been slightly compromised due to the lack of recent advancements in prompt design techniques. The potential impact of prompt design approaches such as chain of thought, reflection, and few-shot in-context learning, which have shown promise in improving model performance, was not evaluated in this study. Consequently, further research is required to validate these prompt design techniques and enhance the accuracy and reliability of AI-assisted classification in medical contexts.”*

**Suggestions (Improvements to Strengthen the Manuscript):**

**Q7. Challenges with ILD and Pneumonia Classification**:
The manuscript should expand on why the models, particularly GPT-4, had lower accuracy in diagnosing conditions like ILD and pneumonia. A discussion about the radiologic features of these diseases and the need for clinical correlation would help contextualize the models’ performance.

**Reply:** Thank you for your insightful feedback. Diagnosing ILD and pneumonia based solely on imaging findings is challenging. ILD can be classified into many different types depending on the cause, clinical presentation, and histological findings. The imaging spectrum is highly variable, including honeycombing, reticular opacity, traction bronchiectasis, fibrosis, ground-glass opacity, consolidation, nodules, and cystic lesions (Ref. 1). However, similar imaging findings, such as fibrosis, ground-glass opacity, and consolidation, can also appear in cases of infection, drug reactions, or radiation therapy. Thus, a comprehensive assessment of the clinical context is essential when confirming ILD. Similarly, pneumonia cannot be diagnosed based solely on imaging findings and must be accompanied by clinical signs and symptoms (Ref. 2). This highlights that LLMs are not yet fully capable of considering clinical correlations, which may explain the reduced accuracy of the LLM models in these conditions.

We analyzed cases categorized by GPT-4 as ILD but diagnosed otherwise by pulmonologists . The most common cases, accounting for 53.7% of these instances, occurred when GPT-4 classified any findings of fibrosis on chest CT, regardless of the cause (e.g., radiotherapy, COVID, tuberculosis), as ILD, whereas pulmonologists did not. Additionally, due to the challenge of diagnosing ILD solely on one finding, pulmonologists did not classify cases of interlobular septal thickening, which could also indicate pulmonary edema or lymphangitic metastasis, as ILD. Conversely, GPT-4 classified them as ILD (22.1%).

We have addressed this in detail in the Discussion section.

**References**

1. Raghu G, Remy-Jardin M, Richeldi L, Thomson CC, Inoue Y, Johkoh T, et al. Idiopathic Pulmonary Fibrosis (an Update) and Progressive Pulmonary Fibrosis in Adults: An Official ATS/ERS/JRS/ALAT Clinical Practice Guideline. Am J Respir Crit Care Med. 2022; 205: e18-e47.

2. Metlay JP, Waterer GW, Long AC, Anzueto A, Brozek J, Crothers K, et al. Diagnosis and Treatment of Adults with Community-acquired Pneumonia. An Official Clinical Practice Guideline of the American Thoracic Society and Infectious Diseases Society of America. Am J Respir Crit Care Med. 2019; 200: e45-e67.

**Changes in the text:** Lines 267–277

*“For ILD, the specificity was relatively low (0.89 in X-ray and 0.88 in chest CT). ILD can be classified into many different types depending on the cause, clinical presentation, and histological findings. The imaging spectrum is highly variable, including honeycombing, reticular opacity, traction bronchiectasis, fibrosis, ground-glass opacity, consolidation, nodules, and cystic lesions [20]. However, similar imaging findings, such as fibrosis, ground-glass opacity, and consolidation, can also appear in cases of infection, drug reactions, or radiation therapy. Thus, a comprehensive assessment of the clinical context is essential when confirming ILD. Similarly, pneumonia cannot be diagnosed based solely on imaging findings and must be accompanied by clinical signs and symptoms [21]. This highlights that LLMs are not yet fully capable of considering clinical correlations, which may explain the reduced accuracy of the LLM models in these conditions.”*

**Q8. Clarification on Data Exclusion Criteria**:
The authors should provide a rationale for excluding reports with minimal findings, such as “no active lung lesions.” Explaining how this exclusion might affect the model’s learning and generalizability will strengthen the study’s methodology section. This can also be added to the limitation section.

**Reply:** We appreciate your concern about the potential bias introduced by excluding cases with minimal findings. We would like to clarify our rationale for this decision and its implications for the study results.

The exclusion of reports with minimal findings, such as “no active lung lesions” or “no significant interval changes,” was a deliberate choice to ensure the quality and clinical relevance of our dataset. In the Korean healthcare context, it is not uncommon to encounter X-ray reports with very limited or no interpretable content. Including such reports could potentially lead to an overestimation of model performance, as they represent overly simplistic tasks that do not reflect the complexity of real-world clinical scenarios.

By focusing on reports with substantial findings, we aimed to challenge our models with more clinically relevant and complex cases, thereby providing a more accurate assessment of their capabilities in real-world settings. This approach ensures that our evaluation of model performance and generalizability is more rigorous and applicable to practical clinical situations where interpretation of significant findings is required.

Regarding the inclusion of normal cases, while they certainly have a place in comprehensive medical imaging datasets, our study specifically aimed to evaluate the models’ performance on cases requiring active interpretation and decision-making. The inclusion of a large number of normal cases might indeed affect the models’ learning, potentially leading to over-prediction of “normal” states, which could be problematic in a clinical setting where missing a significant finding is a critical error.

We acknowledge that this decision may limit the generalizability of our results to all types of radiological reports. However, we believe it provides a more stringent test of the models’ capabilities in scenarios most relevant to clinical decision-making. We have added a note about this limitation in our Discussion section to ensure transparency about the scope and applicability of our findings.

**Changes in the text:** Lines 108–115

*“During the study period, the first radiographic or CT scans of inpatients admitted to the pulmonology department were selected. Reports lacking sufficient or suitable content for interpretation were omitted to ensure the quality and relevance of our dataset, as such reports could lead to an overestimation of model performance by representing overly simplistic tasks that do not reflect the complexity of real-world clinical scenarios. These comprised reports including “no active lung lesions”, “hypoinflated lung”, “no significant interval changes”, “no abnormalities”, “negative chest findings”, “within normal limits”, “simple postoperative status”, and “unremarkable findings”.”*

**Q9. Broader Dataset and Multimodal AI Research**:
Future research should consider incorporating datasets from multiple regions with diverse reporting styles. Additionally, exploring multimodal AI approaches that include image data alongside text could enhance the models’ ability to classify complex conditions like ILD and pneumonia.

**Reply:** Thank you for your valuable feedback. As mentioned in Q6, a limitation of this study is that it analyzed radiology reports written by Korean radiologists in a non-English-speaking country. Future research should aim to validate the model across multiple countries and languages. Ultimately, we hope for a future where AI can classify findings based on the images themselves, rather than relying solely on the reports. These points have been further addressed in the Discussion section.

**Changes in the text:** Lines 346–356

*“Moreover, this research utilized radiologic reports written by Korean radiologists, which may limit its generalizability to regions with different reporting styles and medical practices [28]. Future studies could benefit from validating these findings using datasets from other countries and linguistic backgrounds to assess the models’ performance across diverse contexts. Lastly, the models’ performance in this study may have been slightly compromised due to the lack of recent advancements in prompt design techniques. The potential impact of prompt design approaches such as chain of thought, reflection, and few-shot in-context learning, which have shown promise in improving model performance, was not evaluated in this study. Consequently, further research is required to validate these prompt design techniques and enhance the accuracy and reliability of AI-assisted classification in medical contexts.”*

**Q10. Practical Implementation and Clinical Impact**:
A discussion on how these LLMs could be integrated into clinical workflows, particularly in reducing manual chart reviews without sacrificing diagnostic accuracy, would add practical value to the study

**Reply:** Thank you for the insightful suggestion. Ultimately, we hope that diagnosis and classification through image input alone will significantly reduce the time spent on manual chart reviews, not only for radiologists but also for researchers. This would facilitate large-scale medical research using real-world data. These points have been mentioned in the Discussion.

**Changes in the text:** Lines 357–367

*“In conclusion, this study demonstrated the capability of LLMs, particularly GPT-4, to efficiently extract clinical data from unstructured radiologic reports without the need for additional training in respiratory research. The capability is accompanied by high accuracy, sensitivity, and specificity. These results suggest the potential of automating data extraction processes to improve clinical data repositories and facilitate large-scale medical studies. The proficiency of LLMs, particularly GPT-4, in accurately classifying unstructured radiological data hints at their potential as alternatives to the traditional manual chart reviews conducted by clinicians. However, the variability in performance observed across diseases and healthcare facilities, as well as disparities in human labelling, highlights the need for further research, establishment of standardized guidelines, and comprehensive training to ensure the robustness of models and consistent interpretation of data.”*

**Reviewer 1:**

The authors present an interesting manuscript to use a large language model to extract data on major pulmonary diseases from unstructured radiologic reports. The authors found that GPT-4 was the most accurate.

**Q1.** Regarding statistics, the authors need to explain in explicit detail how sensitivity, specificity, and accuracy were quantified in this study. This is not found in the statistical analysis section.

**Reply:** We appreciate your request for a detailed explanation of our statistical analysis. We acknowledge that our original description lacked specificity regarding the calculation of sensitivity, specificity, and accuracy. To address this, we have added more details to our Methods section. This addition provides a clear explanation of how we quantified these key performance metrics in the context of our study, without resorting to basic definitions that might be considered common knowledge in the field. We believe this clarification strengthens our methodology section and addresses your concern.

**Changes in the text:** Lines 139–149

*“The reports were analyzed using Gemini Pro 1.0, GPT-3.5, and GPT-4, maintaining a consistent temperature of 0.3 across all models. The performance of these models in accurately interpreting reports, compared to the gold standard, was assessed in terms of accuracy, sensitivity, and specificity. For each of the seven diseases and each model (Gemini Pro 1.0, GPT-3.5, and GPT-4), we calculated sensitivity, specificity, and accuracy by comparing the model’s predictions against the gold standard established by agreement between pulmonologists. These metrics were computed separately for each disease category, allowing for a detailed assessment of model performance across different clinical entities. This approach enabled us to evaluate how well each model identified both the presence and absence of specific diseases in the radiologic reports, providing a comprehensive view of their diagnostic capabilities.”*

**Q2.** Is this large language model able to differentiate between acronyms vs. spelled out words (PNA for pneumonia, TB for tuberculosis, etc.)

**Reply:** Thank you for this important question. We would like to clarify that the large language models used in our study (Gemini Pro 1.0, GPT-3.5, and GPT-4) are indeed capable of understanding and differentiating between abbreviations and their full forms in context.

These models are trained on vast amounts of text data, including medical literature, which enables them to recognize common medical abbreviations like PNA for pneumonia and TB for tuberculosis, as well as their spelled-out versions. The models interpret these terms within the context of the entire radiologic report, allowing them to make accurate inferences regardless of whether abbreviations or full terms are used. We did not perform any specific preprocessing to standardize acronym usage, as the models demonstrated the ability to naturally handle this variation. This capability is part of the models’ strength in processing natural language as it appears in real-world medical reports, contributing to their effectiveness in our study.

**Changes in the text:** Lines 303–310

*“Furthermore, the LLM interpreted medical abbreviations within the context of the entire radiologic report, making inferences regardless of whether abbreviations or full terms were used. For instance, GPT-4 correctly recognized all instances of “TB” as “tuberculosis” in the reports. We did not perform any specific preprocessing to standardize acronym usage, as the models demonstrated the ability to handle this variation naturally. These capabilities are part of the models’ strength in processing natural language as it appears in real-world medical reports, contributing to its effectiveness in our study.”*

**Q3.** How does this model account for clinical uncertainty? If a radiologist reads “emphysema vs. COPD” or “lung cancer vs. metastasis”...can the model account for these clinical obscurities as they often required clinical correlation.

**Reply:** We appreciate your valuable comment regarding how LLMs handle clinical uncertainty in radiologic reports. The models we used were pre-trained on various medical literature and seem to avoid making judgments on items with clinical uncertainty, that is, items in the radiologic report lacking definitive evidence for diagnosis. For example, upon reviewing the decisions made by GPT-4, it did not classify “developing and increasing in size of heterogeneously enhancing nodules in the right lower lobe” as lung cancer on a CT scan of a patient with breast cancer. In a case where the report mentioned the possibility of chronic obstructive pulmonary disease—a condition where emphysema may appear in radiologic findings but is not essential for diagnosis—in a patient with chronic bronchitis, GPT-4 did not classify the finding as emphysema.

**Changes in the text:** Lines 295–310

*“On the other hand, in some cases, it appeared that the LLM correlated diagnostic criteria with the context of the radiologic report and avoided making judgments on items with clinical uncertainty, that is, items in the report lacking definitive evidence for diagnosis. For example, upon reviewing the decisions made by GPT-4, it did not classify “developing and increasing in size of heterogeneously enhancing nodules in the right lower lobe” as lung cancer on a CT scan of a patient with breast cancer. In a case where the report mentioned the possibility of chronic obstructive pulmonary disease—a condition where emphysema may appear in radiologic findings but is not essential for diagnosis [24]—in a patient with chronic bronchitis, GPT-4 did not classify the finding as emphysema.”*

**Q4.** What are the qualifications of the interobserver pulmonologists?

**Reply:** Thank you for your valuable comment. The three pulmonologists who performed the labeling have over 10 years each of experience as physicians, including more than 5 years each of clinical experience as pulmonologists. All three hold assistant professorships at university hospitals. GC’s clinical and research areas are tuberculosis (mycobacterial disease), lung cancer, and interventional pulmonology. MGC specializes in lung cancer and interventional pulmonology, and J-YH’s areas of expertise are lung cancer and interstitial lung disease. These details have been added to the Methods section.

**Changes in the text:** Lines 118–131

*“The following outcomes were defined: pneumonia, interstitial lung disease (ILD), active pulmonary tuberculosis (TB), pulmonary edema, pleural effusion, lung cancer, and emphysema. On CT scans, interstitial lung abnormalities were identified as ILD, and lung abscesses were classified as pneumonia. The complete resolution and improvement were recorded as absent, whereas interval improvement and decreases were recorded as present. On chest radiographs, pulmonary congestion was assessed for edema, and interstitial pneumonia was categorized as ILD. Three pulmonology specialists—GC, MGC, and J-YH—each with over a decade of experience as practicing physicians including more than 5 years each of clinical experience in pulmonology independently reviewed the reports and labeled the presence or absence of seven diseases. All three hold assistant professorships at university hospitals with clinical and research expertise. GC’s clinical and research areas include tuberculosis (mycobacterial disease), lung cancer, and interventional pulmonology; MGC specializes in lung cancer and interventional pulmonology; and J-YH’s areas of expertise are lung cancer and interstitial lung disease. The gold standard was established as an agreement among at least two labelers.”*

**Q5.** Does the language model take into account the read of a trainee vs. attending? Perhaps this model can help trainees better understand their shortcomings when over-reads by attendings have different reads or diagnoses.

**Reply:** We appreciate your interesting question regarding the potential application of our model in differentiating between trainee and attending physician reads. However, we would like to clarify that our study focused on analyzing finalized radiologic reports, which typically represent the consensus or final interpretation by attending radiologists.

Our current study design did not include a comparison between trainee and attending physician reads. The reports we used were final interpretations, and we did not have access to or include preliminary reads by trainees in our dataset.

While the potential application of language models in comparing trainee and attending reads is an intriguing idea, it falls outside the scope of our current study. This could indeed be an interesting direction for future research, potentially using our methodology to analyze how language models might assist in medical education and training.

**Q6.** The error rate of 0% from GPT-4 seems impossible as even attending pulmonologists are wrong a small percentage of the time when they read these images. There is also no introduction on JSON format before it is introduced in table 3 and makes it difficult to understand the meaning of that table.

**Reply:** We appreciate your careful observation regarding the reported 0% error rate for GPT-4 and the lack of introduction to JSON formatting. We would like to clarify these points:

Firstly, it’s important to note that the 0% error rate refers specifically to GPT-4’s ability to generate syntactically correct JSON formats, not to its accuracy in disease classification. We have added a clarification in our methods and discussion sections to explain that “error-free” in this context means outputs that strictly adhered to the specified JSON structure, including all seven disease fields with appropriate “yes” or “no” values, without any syntactical errors or missing information. This is distinct from the model’s performance in accurately classifying diseases, which is separately reported in our performance analysis and does indeed show some degree of error, as would be expected.

Regarding the introduction of the JSON format, we acknowledge the oversight in not providing a proper introduction before Table 3. To address this, we have added a brief explanation of JSON formatting and its relevance to our study in the Methods section. The added content explains that the output of each model was evaluated for adherence to the specified JSON format, which included the correct structure and presence of all seven disease fields. This addition provides necessary context for understanding Table 3 and its significance in our study.

We thank you for bringing these points to our attention, allowing us to improve the clarity and comprehensibility of our manuscript.

**Changes in the text:** Lines 325–330

*“Although all AI models demonstrated strong agreement with human annotators in terms of performance, notable discrepancies were observed in their stability when generating JSON formats. GPT-4 showcased exceptional stability, flawlessly producing error-free JSON formats on its first attempt. Error-free JSON formats refer to outputs that strictly adhered to the specified structure, including all seven disease fields with appropriate “yes” or “no” values, without any syntactical errors or missing information.”*

**Changes in the text:** Lines 150–156

*“Additionally, the accuracy of each label was evaluated against the gold standard using these metrics, stratified by disease and hospital, to determine the accuracy and appropriateness of human annotation. Interobserver agreement among the labelers was assessed by calculating the Fleiss kappa value for each disease. The output of each model was evaluated for adherence to the specified JSON format, which included the correct structure and presence of all seven disease fields. Errors in JSON formatting were recorded and analyzed to assess the models’ ability to consistently produce structured output.”*

**Q7.** The more applicable use of AI will be the interpretation of the images themselves vs. just the radiology reports.

**Reply:** Thank you for the valuable feedback. We agree with your opinion and, as discussed in the limitations section of the manuscript, we hope for a future where classification can be done directly from the images themselves, rather than relying on radiology reports.

**Reviewer 2:**

This manuscript presents a compelling study on the application of large language models (LLMs), such as GPT-4, GPT-3.5, and Google’s Gemini Pro, for extracting clinical data on major pulmonary diseases from unstructured radiologic reports. The study’s design, methodology, and results highlight the potential for AI to enhance radiologic data interpretation, addressing a critical challenge in the healthcare field.

****Strengths:****
1. **Novel Application of LLMs**: The study explores a novel use case for LLMs, showing their capacity to accurately interpret radiological reports. This is a valuable addition to the growing literature on AI in healthcare, particularly in contexts where manual review is labor-intensive.
2. **High Accuracy and Specificity**: The results demonstrate that GPT-4, in particular, performed with impressive accuracy (0.89–0.99) and sensitivity across a range of pulmonary conditions, such as pleural effusion and emphysema. This confirms the utility of LLMs in clinical settings, especially for standardizing unstructured data.
3. **Ethical Considerations and Methodology**: The inclusion of ethical approval from multiple institutions and a clear explanation of the retrospective nature of the study, combined with the involvement of experienced pulmonologists, gives the study a robust foundation.

**Reply:** Thank you for your valuable comments. This study attempted to extract and classify clinical data from unstructured radiologic reports using LLM, demonstrating high accuracy. We believe that such research highlights the potential for automating data extraction in large-scale studies in the future.

****Areas for Improvement:****
**Q1.** **Limited External Validation**: The study focuses on radiologic reports written by Korean radiologists, which may limit the generalizability of the findings to other regions where radiologic reporting styles differ significantly. Future research should consider validating these findings on more diverse datasets to assess whether the models perform consistently across languages and cultural differences in medical practice.

**Reply:** Thank you for pointing out the potential limitations regarding external validation of our study. We acknowledge that our research utilized radiologic reports written by Korean radiologists, which may limit its generalizability to other regions where reporting styles and medical practices may differ. As you suggested, future studies could benefit from validating the findings on datasets from different countries and linguistic backgrounds to determine the model’s performance across various contexts. We have added this point to our discussion of the study’s limitations.

**Changes in the text:** Lines 346–356

*“Moreover, this research utilized radiologic reports written by Korean radiologists, which may limit its generalizability to regions with different reporting styles and medical practices [28]. Future studies could benefit from validating these findings using datasets from other countries and linguistic backgrounds to assess the models’ performance across diverse contexts. Lastly, the models’ performance in this study may have been slightly compromised due to the lack of recent advancements in prompt design techniques. The potential impact of prompt design approaches such as chain of thought, reflection, and few-shot in-context learning, which have shown promise in improving model performance, was not evaluated in this study. Consequently, further research is required to validate these prompt design techniques and enhance the accuracy and reliability of AI-assisted classification in medical contexts.”*

**Q2.** **Interpretation Challenges for Specific Conditions**: The models, particularly GPT-4, showed lower performance for diseases like interstitial lung disease (ILD) and pneumonia. It would be beneficial to provide a more in-depth discussion on why these specific conditions pose a challenge for LLMs, potentially incorporating additional medical context.

**Reply:** Thank you for your insightful feedback. It is very challenging to diagnose ILD and pneumonia based solely on imaging findings. ILD can be classified into many different types, depending on the cause, clinical presentation, and histological findings. The imaging spectrum is highly variable, including honeycombing, reticular opacity, traction bronchiectasis, fibrosis, ground-glass opacity, consolidation, nodules, and cystic lesions (Ref. 1). However, similar imaging findings, such as fibrosis, ground-glass opacity, and consolidation, can also appear in cases of infection, drug reactions, or radiation therapy. Thus, a comprehensive assessment of the clinical context is essential when confirming ILD. Similarly, pneumonia cannot be diagnosed based solely on imaging findings and must be accompanied by clinical signs and symptoms (Ref. 2). This highlights that LLMs are not yet fully capable of considering clinical correlations, which may explain the reduced accuracy of the LLM model in these conditions.

We analyzed cases categorized by GPT-4 as ILD but diagnosed otherwise by pulmonologists . The most common cases, accounting for 53.7% of these instances, occurred when GPT-4 classified any findings of fibrosis on chest CT, regardless of the cause (e.g., radiotherapy, COVID, tuberculosis), as ILD, whereas pulmonologists did not. Additionally, due to the challenge of diagnosing ILD solely on one finding, pulmonologists did not classify cases of interlobular septal thickening, which could also indicate pulmonary edema or lymphangitic metastasis, as ILD. Conversely, GPT-4 classified them as ILD (22.1%).

We have addressed this in detail in the Discussion section.

**References**

1. Raghu G, Remy-Jardin M, Richeldi L, Thomson CC, Inoue Y, Johkoh T, et al. Idiopathic Pulmonary Fibrosis (an Update) and Progressive Pulmonary Fibrosis in Adults: An Official ATS/ERS/JRS/ALAT Clinical Practice Guideline. Am J Respir Crit Care Med. 2022; 205: e18-e47.

2. Metlay JP, Waterer GW, Long AC, Anzueto A, Brozek J, Crothers K, et al. Diagnosis and Treatment of Adults with Community-acquired Pneumonia. An Official Clinical Practice Guideline of the American Thoracic Society and Infectious Diseases Society of America. Am J Respir Crit Care Med. 2019; 200: e45-e67.

**Changes in the text:** Lines 267–277

*“For ILD, the specificity was relatively low (0.89 in X-ray and 0.88 in chest CT). ILD can be classified into many different types depending on the cause, clinical presentation, and histological findings. The imaging spectrum is highly variable, including honeycombing, reticular opacity, traction bronchiectasis, fibrosis, ground-glass opacity, consolidation, nodules, and cystic lesions [20]. However, similar imaging findings, such as fibrosis, ground-glass opacity, and consolidation, can also appear in cases of infection, drug reactions, or radiation therapy. Thus, a comprehensive assessment of the clinical context is essential when confirming ILD. Similarly, pneumonia cannot be diagnosed based solely on imaging findings and must be accompanied by clinical signs and symptoms [21]. This highlights that LLMs are not yet fully capable of considering clinical correlations, which may explain the reduced accuracy of the LLM models in these conditions.”*

**Q3.** **Data Limitations**: The exclusion of cases with “no active lung lesions” or “no significant interval changes” may introduce a bias in the dataset. It would be helpful to include a rationale for this decision and how it may affect the study’s overall results. Additionally, clarifying whether the inclusion of normal cases might help improve the models’ learning and prediction capabilities would be useful.

**Reply:** We appreciate your concern about the potential bias introduced by excluding cases with minimal findings. We would like to clarify our rationale for this decision and its implications for the study results.

The exclusion of reports with minimal findings, such as “no active lung lesions” or “no significant interval changes,” was a deliberate choice to ensure the quality and clinical relevance of our dataset. In the Korean healthcare context, X-ray reports with very limited or no interpretable content are often encountered. Including such reports could potentially lead to an overestimation of model performance, as they represent overly simplistic tasks that do not reflect the complexity of real-world clinical scenarios.

By focusing on reports with substantial findings, we aimed to challenge our models with more clinically relevant and complex cases, thereby providing a more accurate assessment of their capabilities in real-world settings. This approach ensures that our evaluation of model performance and generalizability is more rigorous and applicable to practical clinical situations where interpretation of significant findings is required.

Regarding the inclusion of normal cases, while they certainly have a place in comprehensive medical imaging datasets, our study specifically aimed to evaluate the models’ performance on cases requiring active interpretation and decision-making. The inclusion of a large number of normal cases might indeed affect the models’ learning, potentially leading to over-prediction of “normal” states, which could be problematic in a clinical setting where missing a significant finding is a critical error.

We acknowledge that this decision may limit the generalizability of our results to all types of radiological reports. However, we believe it provides a more stringent test of the models’ capabilities in scenarios most relevant to clinical decision-making. We have added a note about this limitation in our Discussion section to ensure transparency about the scope and applicability of our findings.

**Changes in the text:** Lines 108–115

*“During the study period, the first radiographic or CT scans of inpatients admitted to the pulmonology department were selected. Reports lacking sufficient or suitable content for interpretation were omitted to ensure the quality and relevance of our dataset, as such reports could lead to an overestimation of model performance by representing overly simplistic tasks that do not reflect the complexity of real-world clinical scenarios. These comprised reports including “no active lung lesions”, “hypoinflated lung”, “no significant interval changes”, “no abnormalities”, “negative chest findings”, “within normal limits”, “simple postoperative status”, and “unremarkable findings”.”*

****Suggestions for Further Research:****
**Q4.** **Broader Dataset and Multimodal AI**: Incorporating datasets from multiple regions with diverse radiologic reporting styles and languages would provide a more comprehensive understanding of the LLMs’ capabilities and limitations. Furthermore, exploring multimodal AI approaches, which incorporate image data along with text, could enhance the classification of complex conditions like ILD and pneumonia.

**Reply:** Thank you for your valuable feedback. As mentioned in Q1, a limitation of this study is that it analyzed radiology reports written by Korean radiologists in a non-English-speaking country. Future research should aim to validate the model across multiple countries and languages. Ultimately, we hope for a future where AI can classify findings based on the images themselves, rather than relying solely on the reports. These points have been further addressed in the Discussion section.

**Changes in the text:** Lines 346–356

*“Moreover, this research utilized radiologic reports written by Korean radiologists, which may limit its generalizability to regions with different reporting styles and medical practices [28]. Future studies could benefit from validating these findings using datasets from other countries and linguistic backgrounds to assess the models’ performance across diverse contexts. Lastly, the models’ performance in this study may have been slightly compromised due to the lack of recent advancements in prompt design techniques. The potential impact of prompt design approaches such as chain of thought, reflection, and few-shot in-context learning, which have shown promise in improving model performance, was not evaluated in this study. Consequently, further research is required to validate these prompt design techniques and enhance the accuracy and reliability of AI-assisted classification in medical contexts.”*

**Q5.** **Clinical Impact and Practical Implementation**: Future studies could explore the practical application of LLMs in clinical workflows, particularly how they can complement the work of radiologists. Assessing how these models can reduce time spent on manual chart reviews without sacrificing diagnostic accuracy will be critical for real-world implementation.

**Reply:** Thank you for the insightful suggestion. Ultimately, we hope that diagnosis and classification through image input alone will significantly reduce the time spent on manual chart reviews, not only for radiologists but also for researchers. This would facilitate large-scale medical research using real-world data. These points have been mentioned in the Discussion section.

**Changes in the text:** Lines 357–367

*“In conclusion, this study demonstrated the capability of LLMs, particularly GPT-4, to efficiently extract clinical data from unstructured radiologic reports without the need for additional training in respiratory research. The capability is accompanied by high accuracy, sensitivity, and specificity. These results suggest the potential of automating data extraction processes to improve clinical data repositories and facilitate large-scale medical studies. The proficiency of LLMs, particularly GPT-4, in accurately classifying unstructured radiological data hints at their potential as alternatives to the traditional manual chart reviews conducted by clinicians. However, the variability in performance observed across diseases and healthcare facilities, as well as disparities in human labelling, highlights the need for further research, establishment of standardized guidelines, and comprehensive training to ensure the robustness of models and consistent interpretation of data.”*

****Conclusion:****
The manuscript contributes significantly to the literature on AI in radiology, offering valuable insights into the application of LLMs for structured data extraction from unstructured reports. While there are some limitations regarding dataset diversity and the challenges posed by certain conditions, the study lays the groundwork for further advancements in AI-driven healthcare solutions.

**Reply:** Thank you for the valuable comment. Our study proposes the advancement of AI-based data extraction and clinical research in the future and suggests the broader utility of AI applications in the medical field.

**** Minor Revision Suggestions:****
**Q6.** **Expand on the challenges with ILD and pneumonia classification**
**Section: Results (Page 8, Lines 169-170)**
Add more details explaining the reasons behind the lower accuracy in diagnosing interstitial lung disease (ILD) and pneumonia. It would be helpful to mention that both diseases often present with overlapping radiologic features that require clinical correlation for accurate diagnosis. You could also discuss how human expertise still plays a role in diagnosing these complex conditions and why AI models may struggle in such cases.

**Example addition:**
“The lower performance for conditions like ILD and pneumonia could be attributed to the complex and often overlapping radiological features of these diseases. For instance, ILD presents with varied findings such as interlobular septal thickening and fibrosis, which can be easily confused with other conditions like pulmonary edema or post-radiotherapy fibrosis. This emphasizes the need for clinical correlation, which AI models like GPT-4 currently lack, thus affecting their sensitivity and specificity in these cases.”

**Reply:** Thank you for your insightful feedback. As mentioned in Q2, diagnosing ILD and pneumonia based solely on imaging findings is challenging. ILD can be classified into many different types, depending on the cause, clinical presentation, and histological findings. The imaging spectrum is highly variable, including honeycombing, reticular opacity, traction bronchiectasis, fibrosis, ground-glass opacity, consolidation, nodules, and cystic lesions (Ref. 1). However, similar imaging findings, such as fibrosis, ground-glass opacity, and consolidation, can also appear in cases of infection, drug reactions, or radiation therapy. Thus, a comprehensive assessment of the clinical context is essential when confirming ILD. Similarly, pneumonia cannot be diagnosed based solely on imaging findings and must be accompanied by clinical signs and symptoms (Ref. 2). This highlights that LLMs are not yet fully capable of considering clinical correlations, which may explain the reduced accuracy of the LLM model in these conditions.

We have revised these points in the Discussion section according to your suggestion.

**References**

1. Raghu G, Remy-Jardin M, Richeldi L, Thomson CC, Inoue Y, Johkoh T, et al. Idiopathic Pulmonary Fibrosis (an Update) and Progressive Pulmonary Fibrosis in Adults: An Official ATS/ERS/JRS/ALAT Clinical Practice Guideline. Am J Respir Crit Care Med. 2022; 205: e18-e47.

2. Metlay JP, Waterer GW, Long AC, Anzueto A, Brozek J, Crothers K, et al. Diagnosis and Treatment of Adults with Community-acquired Pneumonia. An Official Clinical Practice Guideline of the American Thoracic Society and Infectious Diseases Society of America. Am J Respir Crit Care Med. 2019; 200: e45-e67.

**Changes in the text:** Lines 267–277

*“For ILD, the specificity was relatively low (0.89 in X-ray and 0.88 in chest CT). ILD can be classified into many different types depending on the cause, clinical presentation, and histological findings. The imaging spectrum is highly variable, including honeycombing, reticular opacity, traction bronchiectasis, fibrosis, ground-glass opacity, consolidation, nodules, and cystic lesions [20]. However, similar imaging findings, such as fibrosis, ground-glass opacity, and consolidation, can also appear in cases of infection, drug reactions, or radiation therapy. Thus, a comprehensive assessment of the clinical context is essential when confirming ILD. Similarly, pneumonia cannot be diagnosed based solely on imaging findings and must be accompanied by clinical signs and symptoms [21]. This highlights that LLMs are not yet fully capable of considering clinical correlations, which may explain the reduced accuracy of the LLM models in these conditions.”*

**Q7.** **Clarification on data exclusion criteria**
**Section: Methods (Page 7, Lines 129-132)**
Add a brief explanation as to why reports with “no active lung lesions” or other minimal findings were excluded. This will help address potential concerns about selection bias.

**Example addition:**
“These reports were excluded to focus the analysis on instances where pulmonary diseases were present, thus allowing the models to evaluate complex cases more effectively. While this exclusion helps concentrate the model’s learning, it may also introduce selection bias, and future research should consider including such cases to improve the model’s generalization.”

**Reply:** We appreciate your concern about the potential bias introduced by excluding cases with minimal findings. We would like to clarify our rationale for this decision and its implications for the study results.

The exclusion of reports with minimal findings, such as “no active lung lesions” or “no significant interval changes,” was a deliberate choice to ensure the quality and clinical relevance of our dataset. In the Korean healthcare context, it is not uncommon to encounter X-ray reports with very limited or no interpretable content. Including such reports could potentially lead to an overestimation of model performance, as they represent overly simplistic tasks that do not reflect the complexity of real-world clinical scenarios.

By focusing on reports with substantial findings, we aimed to challenge our models with more clinically relevant and complex cases, thereby providing a more accurate assessment of their capabilities in real-world settings. This approach ensures that our evaluation of model performance and generalizability is more rigorous and applicable to practical clinical situations where interpretation of significant findings is required.

Regarding the inclusion of normal cases, while they certainly have a place in comprehensive medical imaging datasets, our study specifically aimed to evaluate the models’ performance on cases requiring active interpretation and decision-making. The inclusion of a large number of normal cases might indeed affect the models’ learning, potentially leading to over-prediction of “normal” states, which could be problematic in a clinical setting where missing a significant finding is a critical error.

We acknowledge that this decision may limit the generalizability of our results to all types of radiological reports. However, we believe it provides a more stringent test of the models’ capabilities in scenarios most relevant to clinical decision-making. We have added a note about this limitation in our Discussion section to ensure transparency about the scope and applicability of our findings.

**Changes in the text:** Lines 108–115

*“During the study period, the first radiographic or CT scans of inpatients admitted to the pulmonology department were selected. Reports lacking sufficient or suitable content for interpretation were omitted to ensure the quality and relevance of our dataset, as such reports could lead to an overestimation of model performance by representing overly simplistic tasks that do not reflect the complexity of real-world clinical scenarios. These comprised reports including “no active lung lesions”, “hypoinflated lung”, “no significant interval changes”, “no abnormalities”, “negative chest findings”, “within normal limits”, “simple postoperative status”, and “unremarkable findings”.”*

**Q8.** **Include a note on dataset generalizability**
**Section: Discussion (Page 13, Lines 267-270)**
Briefly discuss the limitations of using a dataset that originates solely from Korean radiologists. Mention the need for future validation on a broader scale.

**Example addition:**
“While this study provides a solid foundation for evaluating LLMs in radiology, the dataset’s origin—limited to Korean radiologists—may affect the generalizability of the findings. Different radiology practices and reporting styles across countries may influence the model’s performance. Therefore, validation on more diverse datasets, including reports written in various languages and medical contexts, is necessary to assess the broader applicability of the models.”

**Reply:** Thank you for pointing out the potential limitation of using a dataset that originates solely from Korean radiologists. As mentioned in Q1, we acknowledge that our research utilized radiologic reports written by Korean radiologists, which may limit its generalizability to other regions where reporting styles and medical practices may differ. As you suggested, future studies could benefit from validating the findings on datasets from different countries and linguistic backgrounds to determine the model’s performance across various contexts. We have added this point to the Limitations section.

**Changes in the text:** Lines 346–356

*“* *Moreover, this research utilized radiologic reports written by Korean radiologists, which may limit its generalizability to regions with different reporting styles and medical practices [28]. Future studies could benefit from validating these findings using datasets from other countries and linguistic backgrounds to assess the models’ performance across diverse contexts. Lastly, the models’ performance in this study may have been slightly compromised due to the lack of recent advancements in prompt design techniques. The potential impact of prompt design approaches such as chain of thought, reflection, and few-shot in-context learning, which have shown promise in improving model performance, was not evaluated in this study. Consequently, further research is required to validate these prompt design techniques and enhance the accuracy and reliability of AI-assisted classification in medical contexts.”*

**Q9.** **Clarification on JSON format errors**
**Section: Results (Page 10, Lines 232-237)**
You mention error rates for JSON formatting but do not provide an explanation of the impact of these errors. A brief clarification would help contextualize this finding.

**Example addition:**
“While GPT-4 demonstrated a higher accuracy in producing error-free JSON format, the few errors observed in Gemini Pro 1.0 and GPT-3.5 primarily involved incorrect formatting or incomplete answers for specific diseases. These errors, although minor, highlight the importance of refining model outputs for seamless integration into clinical systems.”

**Reply:** We appreciate your careful observation regarding the reported 0% error rate for GPT-4 and the lack of introduction to JSON formatting. We would like to clarify these points:

Firstly, the 0% error rate refers specifically to GPT-4’s ability to generate syntactically correct JSON formats, not to its accuracy in disease classification. We have added a clarification in our methods and discussion sections to explain that “error-free” in this context means outputs that strictly adhered to the specified JSON structure, including all seven disease fields with appropriate “yes” or “no” values, without any syntactical errors or missing information. This is distinct from the model’s performance in accurately classifying diseases, which is separately reported in our performance analysis and does indeed show some degree of error, as would be expected.

Regarding the introduction of JSON format, we acknowledge the oversight in not providing a proper introduction before Table 3. To address this, we have added a brief explanation of JSON formatting and its relevance to our study in the Methods section. The added content explains that the output of each model was evaluated for adherence to the specified JSON format, which included the correct structure and presence of all seven disease fields. This addition provides necessary context for understanding Table 3 and its significance in our study.

We thank you for bringing these points to our attention, allowing us to improve the clarity and comprehensibility of our manuscript.

**Changes in the text:** Lines 325–330

*“Although all AI models demonstrated strong agreement with human annotators in terms of performance, notable discrepancies were observed in their stability when generating JSON formats. GPT-4 showcased exceptional stability, flawlessly producing error-free JSON formats on its first attempt. Error-free JSON formats refer to outputs that strictly adhered to the specified structure, including all seven disease fields with appropriate “yes” or “no” values, without any syntactical errors or missing information.”*

**Changes in the text:** Lines 150–156

*“Additionally, the accuracy of each label was evaluated against the gold standard using these metrics, stratified by disease and hospital, to determine the accuracy and appropriateness of human annotation. Interobserver agreement among the labelers was assessed by calculating the Fleiss kappa value for each disease. The output of each model was evaluated for adherence to the specified JSON format, which included the correct structure and presence of all seven disease fields. Errors in JSON formatting were recorded and analyzed to assess the models’ ability to consistently produce structured output.”*
